# Breast Tissue Composition and Immunophenotype and Its Relationship with Mammographic Density in Women at High Risk of Breast Cancer

**DOI:** 10.1371/journal.pone.0128861

**Published:** 2015-06-25

**Authors:** Jia-Min B. Pang, David J. Byrne, Elena A. Takano, Nicholas Jene, Lara Petelin, Joanne McKinley, Catherine Poliness, Christobel Saunders, Donna Taylor, Gillian Mitchell, Stephen B. Fox

**Affiliations:** 1 Department of Pathology, Peter MacCallum Cancer Centre, East Melbourne, Victoria, Australia; 2 Familial Cancer Centre, Peter MacCallum Cancer Centre, East Melbourne, Victoria, Australia; 3 Department of Surgical Oncology, Peter MacCallum Cancer Centre, East Melbourne, Victoria, Australia; 4 Department of Pathology, University of Melbourne, Parkville, Melbourne, Victoria, Australia; 5 Sir Peter MacCallum Department of Oncology, University of Melbourne, Parkville, Melbourne, Victoria, Australia; 6 School of Surgery University of Western Australia, Crawley, Perth, Western Australia, Australia; 7 Royal Perth Hospital, Perth, Western Australia, Australia; ACTREC, Tata Memorial Centre, INDIA

## Abstract

**Aim:**

To investigate the cellular and immunophenotypic basis of mammographic density in women at high risk of breast cancer.

**Methods:**

Mammograms and targeted breast biopsies were accrued from 24 women at high risk of breast cancer. Mammographic density was classified into Wolfe categories and ranked by increasing density. The histological composition and immunophenotypic profile were quantified from digitized haematoxylin and eosin-stained and immunohistochemically-stained (ERα, ERβ, PgR, HER2, Ki-67, and CD31) slides and correlated to mammographic density.

**Results:**

Increasing mammographic density was significantly correlated with increased fibrous stroma proportion (rs (22) = 0.5226, p = 0.0088) and significantly inversely associated with adipose tissue proportion (rs (22) = -0.5409, p = 0.0064). Contrary to previous reports, stromal expression of ERα was common (19/20 cases, 95%). There was significantly higher stromal PgR expression in mammographically-dense breasts (p=0.026).

**Conclusions:**

The proportion of stroma and fat underlies mammographic density in women at high risk of breast cancer. Increased expression of PgR in the stroma of mammographically dense breasts and frequent and unexpected presence of stromal ERα expression raises the possibility that hormone receptor expression in breast stroma may have a role in mediating the effects of exogenous hormonal therapy on mammographic density.

## Introduction

Mammographic density is a strong and independent risk factor for breast cancer, reported to exceed all other risk factors apart from age and the presence of mutations in high penetrance breast cancer predisposition genes such as *BRCA1* and *BRCA2*[1]. While heritable factors account for approximately 50–60% of the variance in mammographic density[2,3], other determinants including reproductive history and exogenous hormone use, body mass index (BMI), and importantly, tamoxifen treatment influence mammographic density. Tamoxifen has been shown to reduce mammographic density and breast cancer risk in high risk patients although it is not yet clear if tamoxifen’s effects on breast cancer risk and mammographic density share the same underlying mechanism[4].

Although in the general population, the appearance of mammographic density has been attributed to increased fibroglandular tissue[5–8], only a small number of high risk patients including *BRCA1/2* mutation carriers[9] have been studied, and whether the same observations applies to this group of women more broadly is unknown. In addition, there have been few studies of high-risk women where breast tissue was collected specially for the purpose of investigating mammographic density in contrast to tissue collected for other indications.

Therefore, we have investigated the cellular basis of mammographic density in women at high risk of breast cancer defined by established criteria[10] by assessing histological composition. We further undertook immunophenotypic studies to support the genesis of a hypothesis to explain the underlying molecular basis of a change in mammographic density in patients administered hormonal therapy and selective estrogen receptor modulators (SERMs).

## Materials and Methods

### Patients and Specimens

Women at high risk of breast cancer (as defined by the National Breast and Ovarian Cancer Centre, Australia)[10] with at least one breast unaffected by cancer, normal clinical breast examination and undergoing breast cancer imaging, were recruited through the Peter MacCallum Cancer Centre’s Familial Cancer Centre (Victoria, Australia) and Royal Perth Hospital’s High Risk Breast Clinic (Western Australia, Australia). The study was approved by the Peter MacCallum Cancer Centre Ethics of Human Research Committee (Approval number 08/03) and the Royal Perth Hospital Human Research Ethics Committee (Approval number 2008/085). Exclusion criteria were pregnancy or lactation within 1 year prior to recruitment, current use of oral contraceptive pill (OCP), hormone replacement therapy (HRT), tamoxifen, chemotherapy, and clotting disorders or use of non-steroidal anti-inflammatory drugs (NSAIDs). Participants provided written informed consent to join the study, to undergo mammogram and breast biopsy specifically for this study, and for examination of their mammograms and breast tissue.

Mammograms were taken within 12 months prior to breast biopsy. Breast tissue of the upper outer quadrant of the breast was obtained either as ultrasound-guided core biopsies (n = 9) or as tissue sections (n = 15) taken at prophylactic mastectomy between January 2009 and September 2011 at either Royal Perth Hospital or Peter MacCallum Cancer Centre. The tissue was formalin-fixed, processed and paraffin-embedded (FFPE).

### Assessment of mammographic density

The mammographic density of the breast in the region of the biopsy site was assessed from cranial-caudal mammographic films by one experienced observer (GM). The mammograms were ranked from least to most dense (rank 1 being the least dense mammogram) and also categorized for pattern of density in that region using an adaptation of Wolfe’s classification of mammographic density into N1 (almost no density representing fat predominance), P1 (mainly fat with ductal prominence in portions of the breast), P2 (ductal prominence in more than half of the breast) and DY (general increased parenchymal density) groups[11]; the adaptation refers to using the Wolfe classification in the region of interest rather than a score for the entire breast.

### Immunohistochemistry

Sections (3μm thick) were cut from FFPE blocks and immunohistochemically (IHC) stained for oestrogen receptor alpha (ERα), oestrogen receptor beta (ERβ), progesterone receptor (PgR), human epidermal growth factor receptor 2 (HER2), CD31 and Ki-67. Immunohistochemistry staining methods are detailed in S1 Supplementary Methods [12].

### Image analysis

Haematoxylin and eosin (H&E)-stained and IHC-stained slides were scanned using ScanScope XT (Aperio, Vista, CA, USA) at 20x magnification.

**Fig 1:**
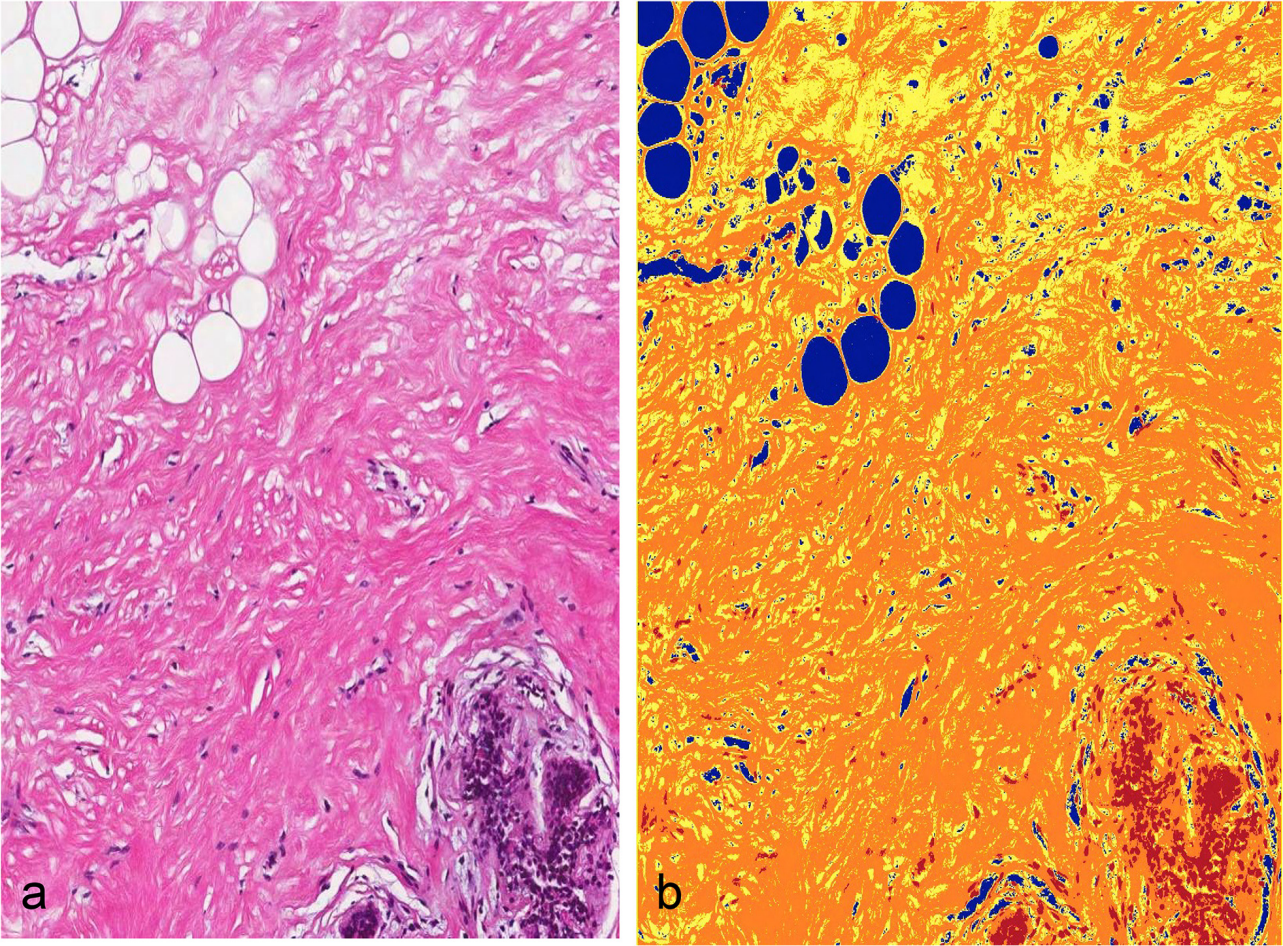
Quantification of proportion of fibrous stroma, fat, and epithelium in breast biopsies. a) H&E-stained section; b) marked-up image of panel a showing strongly staining pixels in red, largely corresponding to epithelium, moderately and weakly staining pixels in orange and yellow respectively, largely corresponding to fibrous stroma, and non-stained pixels in blue, largely corresponding to fat.

The H&E-stained slides were analyzed for tissue composition using the Positive Pixel Count (version 9) image analysis tool (Aperio, Vista, CA, USA). The thresholds for positive staining were chosen after algorithm optimization on H&E-stained sections of archival non-lesional breast tissue from non-study patients and are detailed in S1 Supplementary Methods. From the markup images generated, the number of strongly staining pixels (epithelium), moderate or weak staining pixels (stroma), or negative staining pixels (fat) was used to calculate the proportion of each tissue type from the total number of pixels in the section (Fig 1).

Vascular area was assessed from CD31-stained slides using the Microvascular Analysis Tool, version 1 (Aperio, Vista, CA, USA). The ‘Lumen and Vascular Cells’ analysis option was selected at default settings to generate markup images. The total stained area of the markup images (comprising the vascular lumen in addition to the surrounding endothelial cells) was used to calculate the percentage of vascular area from the total analysis area. (S2 Fig)

**Fig 2:**
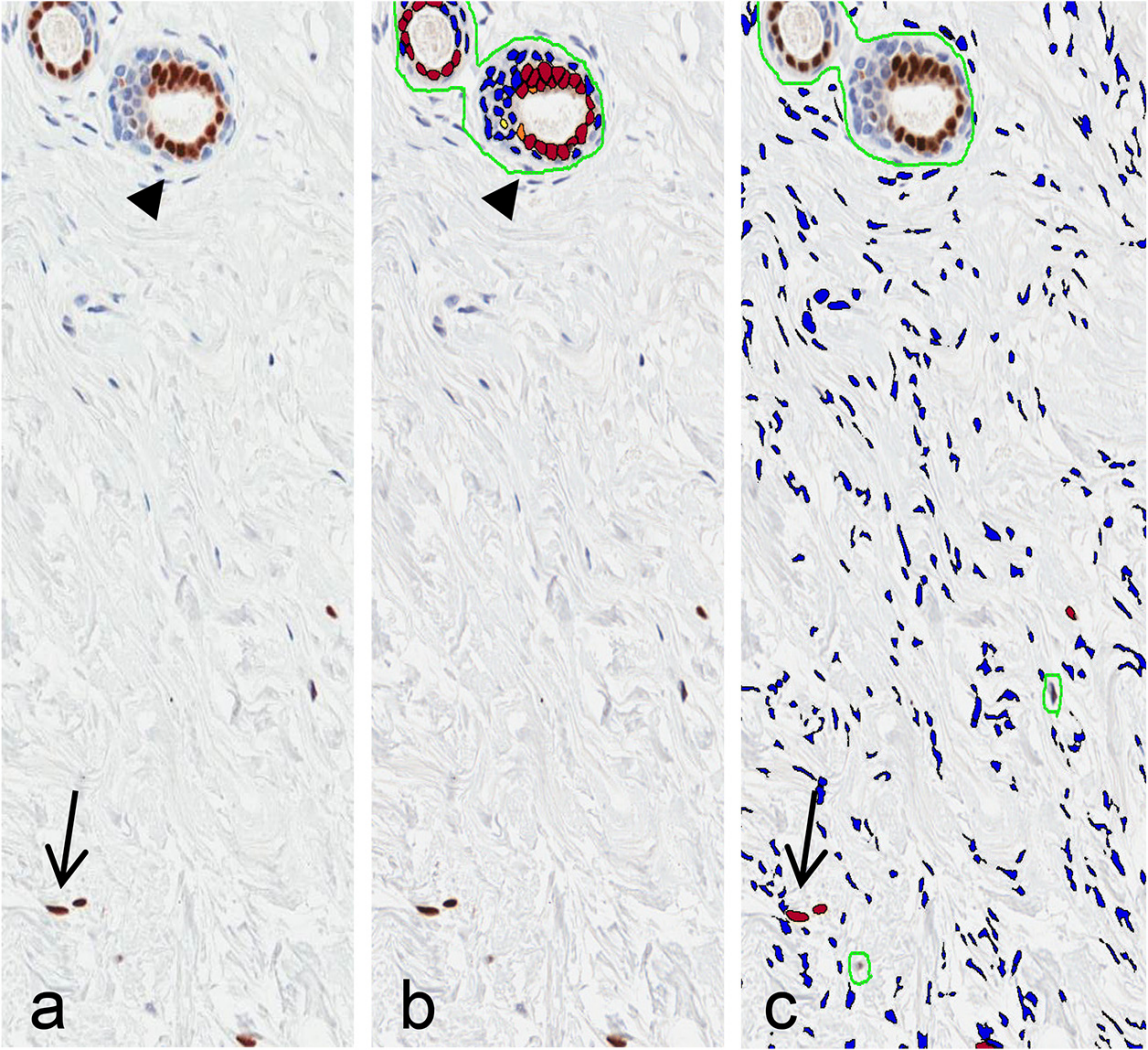
Quantification of IHC staining. a) ERα IHC-stained section with epithelial (filled arrowhead) and stromal (arrow) ERα staining; b) marked-up image of epithelium in panel a showing positive-staining nuclei (filled arrowhead); c) marked-up image of stroma in panel a (epithelium and non-specific staining manually excluded) showing positive-staining nuclei (arrow).

Slides stained for ERα, ERβ, PgR, and Ki-67 were analysed using the Nuclear (version 9) image analysis tool (Aperio, CA, USA) with parameters chosen after algorithm optimization on sections from non-study cases (Fig 2). Epithelial HER2 staining was assessed manually according to the 2013 ASCO/CAP guidelines[13].

### Statistical analysis

Correlations of histological and immunophenotypic features with mammographic density rank and Wolfe pattern categories were evaluated using Spearman’s rank-order correlation and Mann-Whitney U tests, respectively. Due to the small numbers in each Wolfe category, the groups were combined into N1/P1 and P2/DY groups for analysis. Statistical analyses were performed using GraphPad Prism version 6. Two-tailed p-values were used for all analyses with a p-value of less than 0.05 considered statistically significant.

## Results

### Patient flow

A total of 36 patients were recruited into the study. Mammographic films were available for all patients and 32 patients underwent breast biopsy. Three patients were excluded due to current HRT or tamoxifen therapy, two patients were excluded as the side of previous breast cancer was not known and a further two were excluded as the side of biopsy was unknown. One case was excluded as the tissue biopsy was unsuitable for image analysis, leaving 24 cases where both mammogram and biopsy material were available for analysis.

Stromal IHC staining for CD31 and ERα was assessable in 20 cases, Ki-67 in 19 cases, PgR and ERβ in 18 cases. Assessment of epithelial staining of ERα, PgR, Ki-7, and HER2 was possible in 19 cases, and ERβ in 16 cases. The remainder of cases could not be assessed due to poor section quality precluding IHC staining and the absence of epithelium or stroma in the sections.

Patient characteristics are summarized in S1 Table. The median age of the women at time of biopsy was 43 years (range 26–74 years, mean 44 years). Four women (16.7%) and five women (20.8%) had known germline *BRCA1* and *BRCA2* mutations, respectively. Seven women (29.2%) had a history of contralateral breast cancer. Eight women (33.3%) were known to be pre-menopausal and five (20.8%) known to be post-menopausal. Hormonal contraceptives, tamoxifen, and HRT were previously used by eight (33.3%), five (20.8%) and one (4.2%) participants, respectively. All participants ceased exogenous hormone or tamoxifen treatment at least one year prior to inclusion in the study. (S1 Table.)

### Histopathology of biopsies

Nine women underwent breast core biopsies and fifteen women had breast tissue taken from prophylactic mastectomy specimens. The median section size was 11.3mm^2^ for core biopsies (range 2.6–23.4mm^2^), 113.8mm^2^ for tissue taken from mastectomy specimens (range 11.5–458.1mm^2^), and 44.6mm^2^ overall (range 2.6–458.1mm^2^) (S1 Table.). Four biopsies (16.7%) showed benign pathology; two cases showed fibrocystic change and two had ductal hyperplasia of usual type (S1 Table.). No atypical hyperplasia, columnar cell lesions, or malignancy was seen in the biopsies.

### Tissue composition and mammographic density

**Fig 3:**
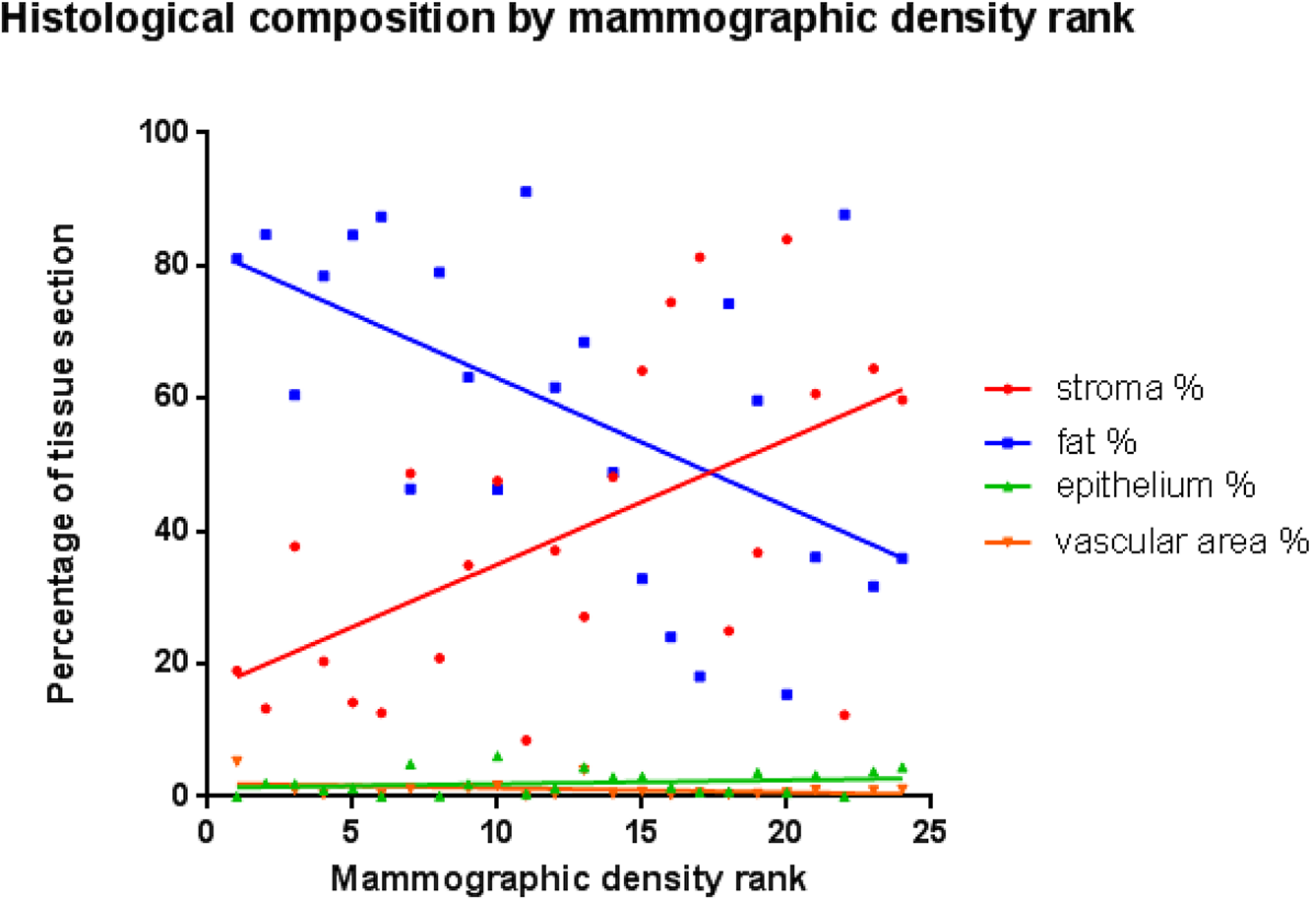
Proportion of fibrous stroma, fat, epithelium, and vascular area by mammographic density rank.

There was a significant positive correlation between increasing mammographic density rank and the proportion of fibrous stroma (rs (22) = 0.5226, p = 0.0088), and a significant inverse relationship between increasing density rank and percentage fat (rs (22) = -0.5409, p = 0.0064) (Table 1, Fig 3). No significant correlation between mammographic density and proportion of epithelium or vascular area (p>0.05) was identified (Table 1) nor between tissue composition and mammographic density as determined by Wolfe categories (p>0.05).

**Table 1:** Histological composition of biopsies.

**Mammographic density rank**		**Tissue component**			
		**Stroma**	**Fat**	**Epithelium**	**Vascular area**
	Spearman’s correlation coefficient	**0.5226**	**-0.5409**	**0.2216**	**-0.1519**
	95% confidence interval	**0.1386 to 0.7700**	**-0.7802 to -0.1635**	**-0.2119 to 0.5822**	**-0.5667 to 0.3243**
	P value	**0.0088**	**0.0064**	**0.2980**	**0.5227**
	Number of pairs	**24**	**24**	**24**	**20**
**Mammographic density Wolfe category**		**Tissue component**			
		**Median percentage stroma**	**Median percentage fat**	**Median percentage epithelium**	**Median percentage vascular area**
	N1/P1	19.00	81.00	1.20	0.99
	P2/DY	47.60	48.90	1.90	0.662
	**Comparison**	**P value**	**P value**	**P value**	**P value**
	N1/P1 vs P2/DY	0.0591	0.0632	0.3755	0.5456

### Stromal and epithelial immunophenotype and mammographic density

**Fig 4:**
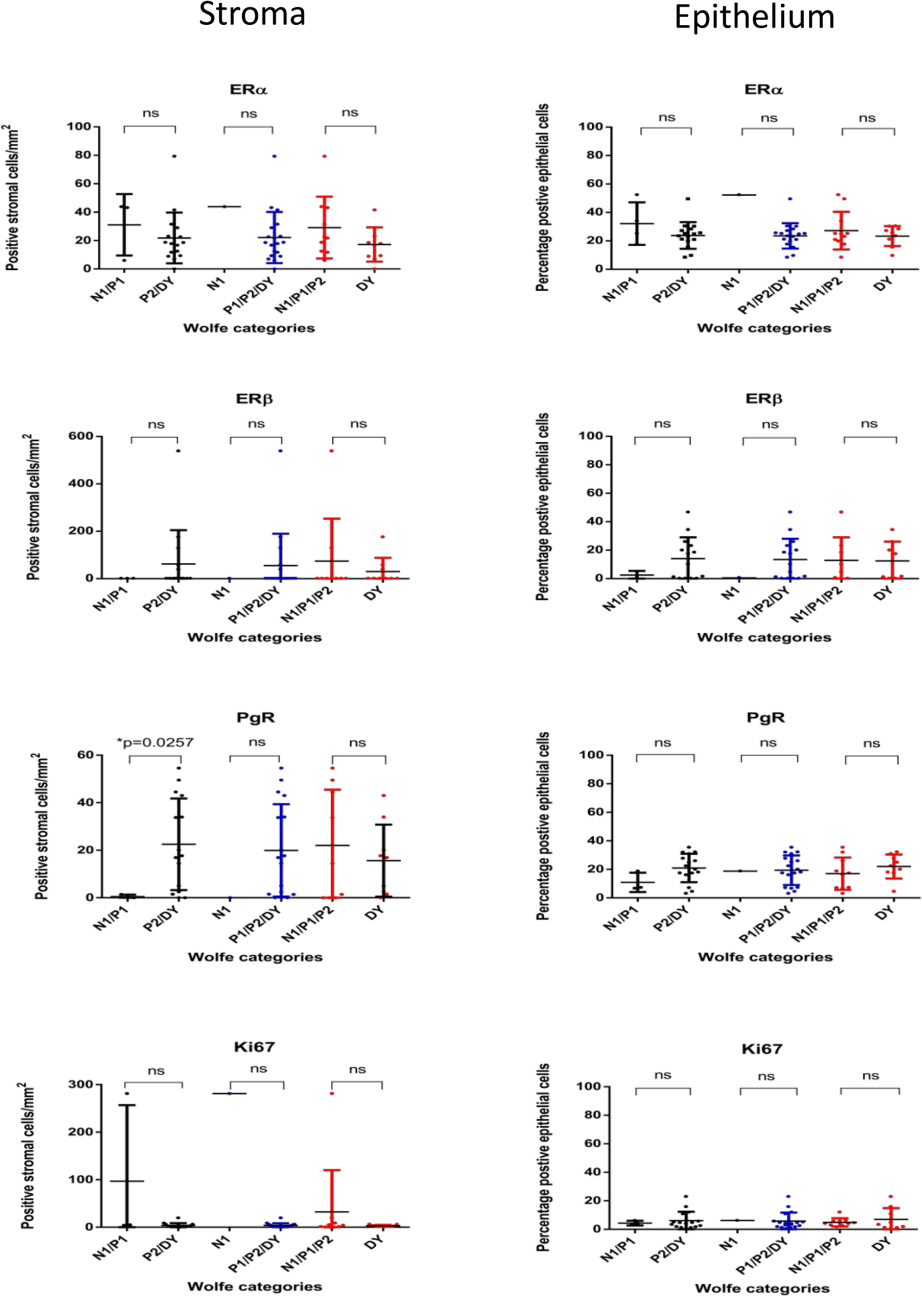
Stromal and epithelial immunophenotype and mammographic density by Wolfe categories. *indicates statistically significant difference, ns = not statistically significant.

Stromal ERα, ERβ, PgR, and Ki-67 staining was present in 95% (19/20), 27.8% (5/18), 77.8% (14/18) and 94.7% (18/19) of cases, respectively. The median stromal expression of ERα, ERβ, PgR, and Ki-67 was 18.7 positive cells/mm^2^ (range 0–79.4/mm^2^), 0/mm^2^ (range 0–538.1/mm^2^), 15.7/mm^2^ (range 0–54.6/mm^2^), and 2.1/mm^2^ (range 0-281/mm^2^), respectively. ERα, PgR and Ki-67 showed moderate to strong intensity staining, whereas ERβ stromal staining was weak. There was no stromal HER2 staining (Fig 3). Significantly higher stromal PgR expression was identified in the denser Wolfe categories (P2/DY) compared with less dense Wolfe categories (N1/P1) (p = 0.0257) but there was no significant correlation between stromal PgR expression and mammographic density rank (p >0.05) (Table 2, Fig 4). No significant correlation was found between the other stromal markers and mammographic density (Table 2, Fig 4), nor between the level of expression of the individual immunohistochemical markers (p>0.05, data not shown).

**Table 2:** Stromal expression of IHC markers.

		IHC marker			
		ERα	ERβ	PgR	Ki-67
**Mammographic density by rank**	Spearman’s correlation coefficient	-0.1940	0.1894	0.0923	-0.0860
	95% confidence interval	-0.5954 to 0.2849	-0.3180 to 0.6125	-0.4041 to 0.5468	-0.5305 to 0.3956
	P value	0.4125	0.4517	0.7156	0.7264
	Number of pairs	20	18	18	19
**Mammographic density by Wolfe category**		Median positive cells/mm^2^ (n)	Median positive cells/mm^2^ (n)	Median positive cells/mm^2^ (n)	Median positive cells/mm^2^ (n)
	N1/P1	43.24 (3)	0 (3)	0 (3)	5.05 (3)
	P2/DY	18.6 (17)	0 (15)	17.65 (15)	1.79 (16)
	N1/P1 vs P2/DY p value	0.4789	0.5221	**0.0257**	0.0846

ERα, ERβ, PgR, and Ki-67-positive epithelial cells were present in 100% (19/19), 75% (12/16), 100% (19/19), and 100% (19/19) of cases, respectively. The median epithelial expression of ERα, ERβ, PgR, and Ki-67 was 24.7% (range 8.3–52.3%), 7.3% (range 0–46.6%), 18.8% (range 3.2–35.4%) and 4.1% (range 0.5–23.0%), respectively. ERα, PgR, and Ki-67 showed predominantly moderate to strong staining, whereas ERβ staining was weak. Epithelial HER2 staining was focal (<10% of cells) and weak where present, which would score as IHC 0 according to the 2013 ASCO/CAP guidelines[13]. No significant correlation was present between mammographic density and epithelial immunophenotype (p>0.05) (Fig 4). Epithelial ERα expression showed a significant positive correlation with epithelial PgR expression (rs (17) = 0.5386, p = 0.0174), but no significant correlation was identified between the expression of the other immunohistochemical markers (p>0.05, data not shown).

## Discussion

This study examined the tissue composition and immunophenotypic profile of ERα, ERβ, PgR, HER2 and Ki-67 in tissue samples taken from clinically and radiologically normal breasts of women at high risk of breast cancer and assessed the relationship of these parameters with mammographic density. This cohort included four women with germline *BRCA1* and five women with *BRCA2* mutations, however meaningful subgroup analysis of these women was not possible due to small numbers. A significant positive correlation between mammographic density and the proportion of fibrous stroma and conversely, a significant inverse correlation between mammographic density and proportion of fat was observed without any significant difference in epithelial or vascular area. It is possible that there is differential shrinkage between fat and non-fatty tissue during histological processing which might influence the ratio of cellular composition but all samples would presumably be similarly affected and therefore this should not unduly bias the results. Furthermore our findings are in keeping with a previous study which examined the association of tissue composition with mammographic density in high breast cancer risk patients undergoing risk-reducing mastectomy, including nine *BRCA1* and *BRCA2* mutation carriers [9]. Our tissue composition findings are also in accordance with those of studies involving non-high risk patients, where high mammographic density was also reported to be associated with increased stroma[6,8,14] and less fat[6,8,9].

The results of studies examining the contribution of epithelial tissue to the appearance of mammographic density are mixed. While some studies have reported no difference in the epithelial component between high and low mammographic dense breasts[7,9], other studies have reported an increased epithelial component in mammographically dense breasts[6,8,15].

The findings of our study and others suggest that the underlying cellular basis of mammographic density is similar in high risk women and the general population.

We also identified a significant association between stromal PgR expression and increased mammographic density, but not for other stromal or epithelial markers. There has been a single study quantifying steroid receptor expression in breast stroma in relation to mammographic density involving 66 patients undergoing mastectomy for breast cancer which reported no significant increase in PgR or ER in dense versus non-dense breasts using the Allred scoring system[16]. It is possible that differences in the scoring system utilized may account for the discrepancy between this study and ours or alternatively that there is a true difference between high risk women without cancer and patients with breast cancer. Mammographic density increases in the luteal phase of the menstrual cycle[17] and with estrogen-progestin combined HRT compared with estrogen alone[18–20], suggesting progesterone has a role in determining mammographic density and recently, it has been hypothesized that reduced progesterone levels may explain the lower risk of breast cancer in obese premenopausal women[21].

The strong and consistent presence of ERα but not ERβ staining in breast stroma of our study cases was confirmed in additional cases of both normal and cancer-containing breast tissue obtained from the pathology department at Peter MacCallum Cancer Centre (data not shown). The presence of stromal ERα was unexpected as previous reports indicate that ERα was absent in breast stroma of adult women[22–24], being limited to occasional cells in children[25,26], teenagers and pregnant women[27]. The detection of stromal ERα in our samples could be possibly be explained by the use of SP1 clone of anti-ERα antibody, since previous studies reporting the absence of ERα in breast stroma of adult women have used clones 6F11[22] and ID5[24]. The SP1 clone is reported to have increased affinity for ERα compared with the ID5 clone[28] and to be more sensitive for the detection of ERα compared with ID5 and 6F11[29,30].

Although we did not identify an association between ERα expression and mammographic density, the finding of stromal ERα expression, whether or not it is explained by the test methodology, raises the hypothesis that stromal ERα may have a role in mediating breast density changes that occurs with administration with hormonal therapy. Cuzick *et al*.[4] reported a reduction in mammographic density and breast cancer risk in moderate and high-risk patients treated with tamoxifen, with the risk reduction occurring in patients with at least 10% reduction in mammographic density[4]. Mammographic density reduction in breast cancer patients receiving adjuvant endocrine therapy has also been reported to be associated with recurrence-free survival[31,32] and lower risk breast cancer-related death[33,34].

Further studies examining the relationship between stromal hormone receptor expression and changes in mammographic density in response to exogenous hormones are required to test our hypothesis.

Although our study numbers are relatively small (n = 24), this is still one of the largest cohorts of *BRCA1/2* carriers (n = 9) in which tissue composition associated with mammographic density has been studied, and the only study to assess mammographic density in living high risk women and to relate this to both histological composition and immunophenotypic profile of ERα, ERβ, PgR, Ki-67 and HER2 in non-tumoural breast tissue. Finally, biopsies were taken from pre-specified target areas that could be correlated with mammographic location.

## Conclusions

Similar to the general population, the proportion of stroma and fat in breast tissue underlies the degree of mammographic density in our cohort of women at high risk of breast cancer. Increased expression of PgR in the stroma of mammographically dense breasts and frequent and unexpected presence of stromal ERα expression raises the hypothesis that hormone receptor expression in breast stroma may have a role in mediating the effects of exogenous hormonal therapy on mammographic density, and requires further investigation beyond the scope of this current study.

## Supporting Information

S1 Table Patient and biopsy characteristics. (DOC)

S2 Table Distribution of IHC markers. (DOC)

S3 Table Epithelial expression of IHC markers. (DOC)

S1 Fig Patient flow in study. (TIF)

S2 Fig Quantification of vascular area in breast biopsies. a) CD31 IHC-stained section; b) marked-up image of panel a with vascular luminal area (gray) and endothelial cells (red) highlighted
(TIF)

S3 Fig Patterns of IHC staining. a) ERβ, b) PgR, c) Ki-67, d) HER2.
(TIF)

S1 Supplementary Methods IHC staining methods and image analysis methods. (DOCX)

## References

[1] Ginsburg OM, Martin LJ, Boyd NF (2008) Mammographic density, lobular involution, and risk of breast cancer. Br J Cancer 99: 1369–1374. 10.1038/sj.bjc.6604635 18781174 PMC2579686

[2] Ursin G, Lillie EO, Lee E, Cockburn M, Schork NJ, Cozen W, et al (2009) The relative importance of genetics and environment on mammographic density. Cancer Epidemiol Biomarkers Prev 18: 102–112. 10.1158/1055-9965.EPI-07-2857 19124487

[3] Boyd NF, Dite GS, Stone J, Gunasekara A, English DR, McCredie MR, et al (2002) Heritability of mammographic density, a risk factor for breast cancer. N Engl J Med 347: 886–894. 12239257 10.1056/NEJMoa013390

[4] Cuzick J, Warwick J, Pinney E, Duffy SW, Cawthorn S, Howell A, et al (2011) Tamoxifen-induced reduction in mammographic density and breast cancer risk reduction: a nested case-control study. J Natl Cancer Inst 103: 744–752. 10.1093/jnci/djr079 21483019

[5] Li T, Sun L, Miller N, Nicklee T, Woo J, Hulse-Smith L, et al (2005) The association of measured breast tissue characteristics with mammographic density and other risk factors for breast cancer. Cancer Epidemiol Biomarkers Prev 14: 343–349. 15734956 10.1158/1055-9965.EPI-04-0490

[6] Ghosh K, Brandt KR, Reynolds C, Scott CG, Pankratz VS, Riehle DL, et al (2012) Tissue composition of mammographically dense and non-dense breast tissue. Breast Cancer Res Treat 131: 267–275. 10.1007/s10549-011-1727-4 21877142 PMC3707294

[7] Alowami S, Troup S, Al-Haddad S, Kirkpatrick I, Watson PH (2003) Mammographic density is related to stroma and stromal proteoglycan expression. Breast Cancer Res 5: R129–135. 12927043 10.1186/bcr622 PMC314426

[8] Vachon CM, Sasano H, Ghosh K, Brandt KR, Watson DA, Reynolds C, et al (2011) Aromatase immunoreactivity is increased in mammographically dense regions of the breast. Breast Cancer Res Treat 125: 243–252. 10.1007/s10549-010-0944-6 20526739 PMC2997154

[9] Lin SJ, Cawson J, Hill P, Haviv I, Jenkins M, Hopper JL, et al (2011) Image-guided sampling reveals increased stroma and lower glandular complexity in mammographically dense breast tissue. Breast Cancer Res Treat 128: 505–516. 10.1007/s10549-011-1346-0 21258862

[10] National Breast and Ovarian Cancer Centre (2010). Advice about familial aspects of breast cancer and epithelial ovarian cancer- a guide for health professionals.

[11] Wolfe JN (1976) Risk for breast cancer development determined by mammographic parenchymal pattern. Cancer 37: 2486–2492. 1260729 10.1002/1097-0142(197605)37:5<2486::aid-cncr2820370542>3.0.co;2-8

[12] Yan M, Rayoo M, Takano EA, Fox SB (2011) Nuclear and cytoplasmic expressions of ERβ1 and ERβ2 are predictive of response to therapy and alters prognosis in familial breast cancers. Breast Cancer Research and Treatment 126: 395–405. 10.1007/s10549-010-0941-9 20490651

[13] Wolff AC, Hammond ME, Hicks DG, Dowsett M, McShane LM, Allison KH, et al (2013) Recommendations for human epidermal growth factor receptor 2 testing in breast cancer: American Society of Clinical Oncology/College of American Pathologists clinical practice guideline update. J Clin Oncol 31: 3997–4013. 10.1200/JCO.2013.50.9984 24101045

[14] Harvey JA, Santen RJ, Petroni GR, Bovbjerg VE, Smolkin ME, Sheriff FS, et al (2008) Histologic changes in the breast with menopausal hormone therapy use: correlation with breast density, estrogen receptor, progesterone receptor, and proliferation indices. Menopause 15: 67–73. 17558338 10.1097/gme.0b013e318054e29a PMC4567838

[15] Hawes D, Downey S, Pearce CL, Bartow S, Wan P, Pike MC, et al (2006) Dense breast stromal tissue shows greatly increased concentration of breast epithelium but no increase in its proliferative activity. Breast Cancer Res 8: R24 16646977 10.1186/bcr1408 PMC1557710

[16] Yang WT, Lewis MT, Hess K, Wong H, Tsimelzon A, Karadag N, et al (2010) Decreased TGFbeta signaling and increased COX2 expression in high risk women with increased mammographic breast density. Breast Cancer Res Treat 119: 305–314. 10.1007/s10549-009-0350-0 19241157 PMC5921048

[17] Kuhl H, Schneider HP (2013) Progesterone—promoter or inhibitor of breast cancer. Climacteric 16 Suppl 1: 54–68. 10.3109/13697137.2013.768806 23336704

[18] Lundstrom E, Christow A, Kersemaekers W, Svane G, Azavedo E, Söderqvist G, et al (2002) Effects of tibolone and continuous combined hormone replacement therapy on mammographic breast density. Am J Obstet Gynecol 186: 717–722. 11967497 10.1067/mob.2002.121896

[19] Greendale GA, Reboussin BA, Slone S, Wasilauskas C, Pike MC, Ursin G (2003) Postmenopausal hormone therapy and change in mammographic density. J Natl Cancer Inst 95: 30–37. 12509398 10.1093/jnci/95.1.30

[20] Vachon CM, Sellers TA, Vierkant RA, Wu FF, Brandt KR (2002) Case-control study of increased mammographic breast density response to hormone replacement therapy. Cancer Epidemiol Biomarkers Prev 11: 1382–1388. 12433715

[21] Dowsett M, Folkerd E (2015) Reduced progesterone levels explain the reduced risk of breast cancer in obese premenopausal women: a new hypothesis. Breast Cancer Res Treat 149: 1–4. 10.1007/s10549-014-3211-4 25414027

[22] Jensen EV, Cheng G, Palmieri C, Saji S, Makela S, van Noorden S, et al (2001) Estrogen receptors and proliferation markers in primary and recurrent breast cancer. Proc Natl Acad Sci U S A 98: 15197–15202. 11734621 10.1073/pnas.211556298 PMC65006

[23] Speirs V, Skliris GP, Burdall SE, Carder PJ (2002) Distinct expression patterns of ER alpha and ER beta in normal human mammary gland. J Clin Pathol 55: 371–374. 11986344 10.1136/jcp.55.5.371 PMC1769648

[24] Palmieri C, Saji S, Sakaguchi H, Cheng G, Sunters A, O’Hare MJ, et al (2004) The expression of oestrogen receptor (ER)-beta and its variants, but not ERalpha, in adult human mammary fibroblasts. J Mol Endocrinol 33: 35–50. 15291741 10.1677/jme.0.0330035

[25] Boyd M, Hildebrandt RH, Bartow SA (1996) Expression of the estrogen receptor gene in developing and adult human breast. Breast Cancer Res Treat 37: 243–251. 8825136 10.1007/BF01806506

[26] Keeling JW, Ozer E, King G, Walker F (2000) Oestrogen receptor alpha in female fetal, infant, and child mammary tissue. J Pathol 191: 449–451. 10918221 10.1002/1096-9896(2000)9999:9999<::AID-PATH661>3.0.CO;2-#

[27] Koerner F, Oyama T, Kurosumi M, Maluf H (2001) Ovarian hormone receptors in human mammary stromal cells. J Steroid Biochem Mol Biol 78: 285–290. 11595509 10.1016/s0960-0760(01)00095-4

[28] Huang Z, Zhu W, Szekeres G, Xia H (2005) Development of new rabbit monoclonal antibody to estrogen receptor: immunohistochemical assessment on formalin-fixed, paraffin-embedded tissue sections. Appl Immunohistochem Mol Morphol 13: 91–95. 15722800 10.1097/00129039-200503000-00015

[29] Bae YK, Gong G, Kang J, Lee A, Cho EY, Lee JS, et al (2012) Hormone receptor expression in invasive breast cancer among Korean women and comparison of 3 antiestrogen receptor antibodies: a multi-institutional retrospective study using tissue microarrays. Am J Surg Pathol 36: 1817–1825. 10.1097/PAS.0b013e318267b012 23154769

[30] Cheang MC, Treaba DO, Speers CH, Olivotto IA, Bajdik CD, Chia SK, et al (2006) Immunohistochemical detection using the new rabbit monoclonal antibody SP1 of estrogen receptor in breast cancer is superior to mouse monoclonal antibody 1D5 in predicting survival. J Clin Oncol 24: 5637–5644. 17116944 10.1200/JCO.2005.05.4155

[31] Ko KL, Shin IS, You JY, Jung SY, Ro J, Lee ES (2013) Adjuvant tamoxifen-induced mammographic breast density reduction as a predictor for recurrence in estrogen receptor-positive premenopausal breast cancer patients. Breast Cancer Res Treat 142: 559–567. 24233999 10.1007/s10549-013-2726-4

[32] Kim J, Han W, Moon HG, Ahn S, Shin HC, You JM, et al (2012) Breast density change as a predictive surrogate for response to adjuvant endocrine therapy in hormone receptor positive breast cancer. Breast Cancer Res 14: R102 10.1186/bcr3221 22770227 PMC3680951

[33] Nyante SJ, Sherman ME, Pfeiffer RM, Berrington de Gonzalez A, Brinton LA, Aiello Bowles EJ, et al (2015) Prognostic Significance of Mammographic Density Change after Initiation of Tamoxifen for ER-Positive Breast Cancer. J Natl Cancer Inst 107. 10.1093/jnci/dju425 PMC4334825 25663687

[34] Li J, Humphreys K, Eriksson L, Edgren G, Czene K, Hall P (2013) Mammographic density reduction is a prognostic marker of response to adjuvant tamoxifen therapy in postmenopausal patients with breast cancer. J Clin Oncol 31: 2249–2256. 10.1200/JCO.2012.44.5015 23610119 PMC3677838

