# Supplementary material for: Breast Tissue Composition and Immunophenotype and Its Relationship with Mammographic Density in Women at High Risk of Breast Cancer
Source: PLoS One. 2015 Jun 25;10(6):e0128861. doi: 10.1371/journal.pone.0128861 (PMC4481506; doi:10.1371/journal.pone.0128861)
Supplement: S1 Table — (DOC) [file pone.0128861.s001.doc]

**S1 Table. Patient and biopsy characteristics.**

| Age | Range  Median  Mean | 26-74 years  43 years  44 years |
| --- | --- | --- |
| *BRCA1/2* mutation status | *BRCA1* mutation  *BRCA2* mutation  Unknown mutation status | 4  5  15 |
| Body mass index (BMI) | Range  Median  Mean  No data | 18.3-41.0 kg/m^2^  27.2 kg/m^2^  28.0 kg/m^2^  12 |
| Menopausal status | Post-menopausal  Pre-menopausal  No data | 5  8  11 |
| Breast cancer history | Previous contralateral breast cancer  No history of breast cancer | 7  17 |
| Age of menarche | Range  Median  Mean | 11-15 years  13 years  12.7 years |
| Parity | 0  1  2  3  4 | 14  2  5  2  1 |
| Age of first birth | Range  Median  Mean | 17-36 years  26.5 years  25.6 years |
| Hormone replacement therapy (HRT) | Previous use  Never used | 1  23 |
| Hormonal contraceptives | Previous use  Never used | 8  16 |
| Tamoxifen | Previous use  Never used | 5  19 |
| Mammographic density by Wolfe classification | N1  P1  P2  DY | 7  2  3  12 |
| Size of biopsies | All biopsies (n=24)  Range  Median  Mean  Core biopsies (n=9)  Range  Median  Mean  Excision biopsies (n=15)  Range  Median  Mean | 2.6-458.1mm^2^  44.6mm^2^  116.7mm^2^  2.6-23.4mm^2^  11.3mm^2^  12.0mm^2^  11.5-458.1mm^2^  113.8mm^2^  179.6mm^2^ |
| Pathology | Fibrocystic change  Ductal hyperplasia of usual type  No pathology | 2  2  20 |
