# Supplementary material for: Breast Tissue Composition and Immunophenotype and Its Relationship with Mammographic Density in Women at High Risk of Breast Cancer
Source: PLoS One. 2015 Jun 25;10(6):e0128861. doi: 10.1371/journal.pone.0128861 (PMC4481506; doi:10.1371/journal.pone.0128861)
Supplement: S2 Table — (DOC) [file pone.0128861.s002.doc]

**S2 Table. Distribution of IHC markers.**

|  | | **IHC marker** | | | |
| --- | --- | --- | --- | --- | --- |
|  |  | **ERα** | **ERβ** | **PgR** | **Ki-67** |
| **Stroma** | Number of cases with stromal expression (%) | 19/20 (95) | 5/18 (27.8) | 14/18 (77.8) | 18/19 (94.7) |
|  | Positive cells/mm2 range | 0-79.4 | 0-538.1 | 0-54.6 | 0-281 |
|  | Positive cells/mm2 median | 18.7 | 0 | 15.7 | 2.1 |
|  |  |  |  |  |  |
| **Epithelium** | Number of cases with epithelial expression (%) | 19/19 (100) | 12/16 (75) | 19/19 (100) | 19/19 (100) |
|  | Percentage positive cells range | 8.3-52.3 | 0-46.6 | 3.2-35.4 | 0.5-23.0 |
|  | Percentage positive cells median | 24.7 | 7.3 | 18.8 | 4.1 |
