# Supplementary material for: Breast Tissue Composition and Immunophenotype and Its Relationship with Mammographic Density in Women at High Risk of Breast Cancer
Source: PLoS One. 2015 Jun 25;10(6):e0128861. doi: 10.1371/journal.pone.0128861 (PMC4481506; doi:10.1371/journal.pone.0128861)
Supplement: S3 Table — (DOC) [file pone.0128861.s003.doc]

**S3 Table. Epithelial expression of IHC markers.**

|  | | **IHC marker** | | | |
| --- | --- | --- | --- | --- | --- |
|  |  | **ERα** | **ERβ** | **PgR** | **Ki-67** |
| **Mammographic density by rank** | Spearman’s correlation coefficient | -0.0684 | 0.0148 | 0.4150 | -0.1203 |
|  | 95% confidence interval | -0.5177 to 0.4104 | -0.4968 to 0.5187 | -0.0478 to 0.7312 | -0.5444 to 0.3528 |
|  | P value | 0.7808 | 0.9582 | 0.0688 | 0.6134 |
|  | Number of pairs | 19 | 16 | 20 | 20 |
|  | | Median % positive epithelial cells (n) | Median % positive epithelial cells (n) | Median % positive epithelial cells (n) | Median % positive epithelial cells (n) |
| **Mammographic density by Wolfe category** | N1/P1 | 25.00 (3) | 2.58 (2) | 7.27 (3) | 4.11 (3) |
|  | P2/DY | 24.02 (16) | 13.71 (14) | 21.21 (16) | 4.28 (16) |
|  |  |  |  |  |  |
|  | Comparison | P value | P value | P value | P value |
|  | N1/P1 vs P2/DY | 0.7121 | 0.5833 | 0.1837 | 0.8751 |
