# Supplementary figures and images for: Breast Tissue Composition and Immunophenotype and Its Relationship with Mammographic Density in Women at High Risk of Breast Cancer
Source: PLoS One. 2015 Jun 25;10(6):e0128861. doi: 10.1371/journal.pone.0128861 (PMC4481506; doi:10.1371/journal.pone.0128861)

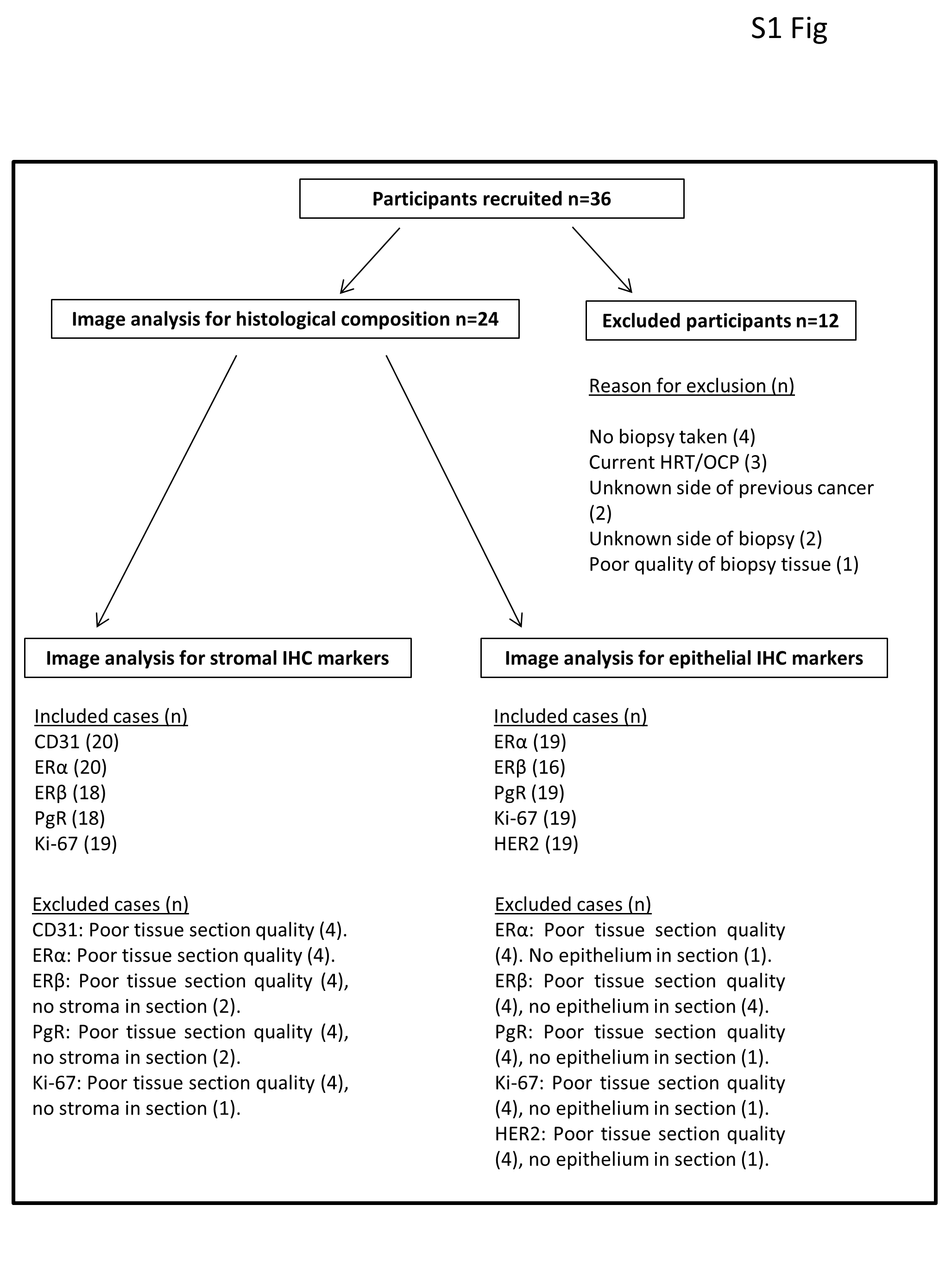

Supplement: S1 Fig — (TIF) [file pone.0128861.s004.tif]

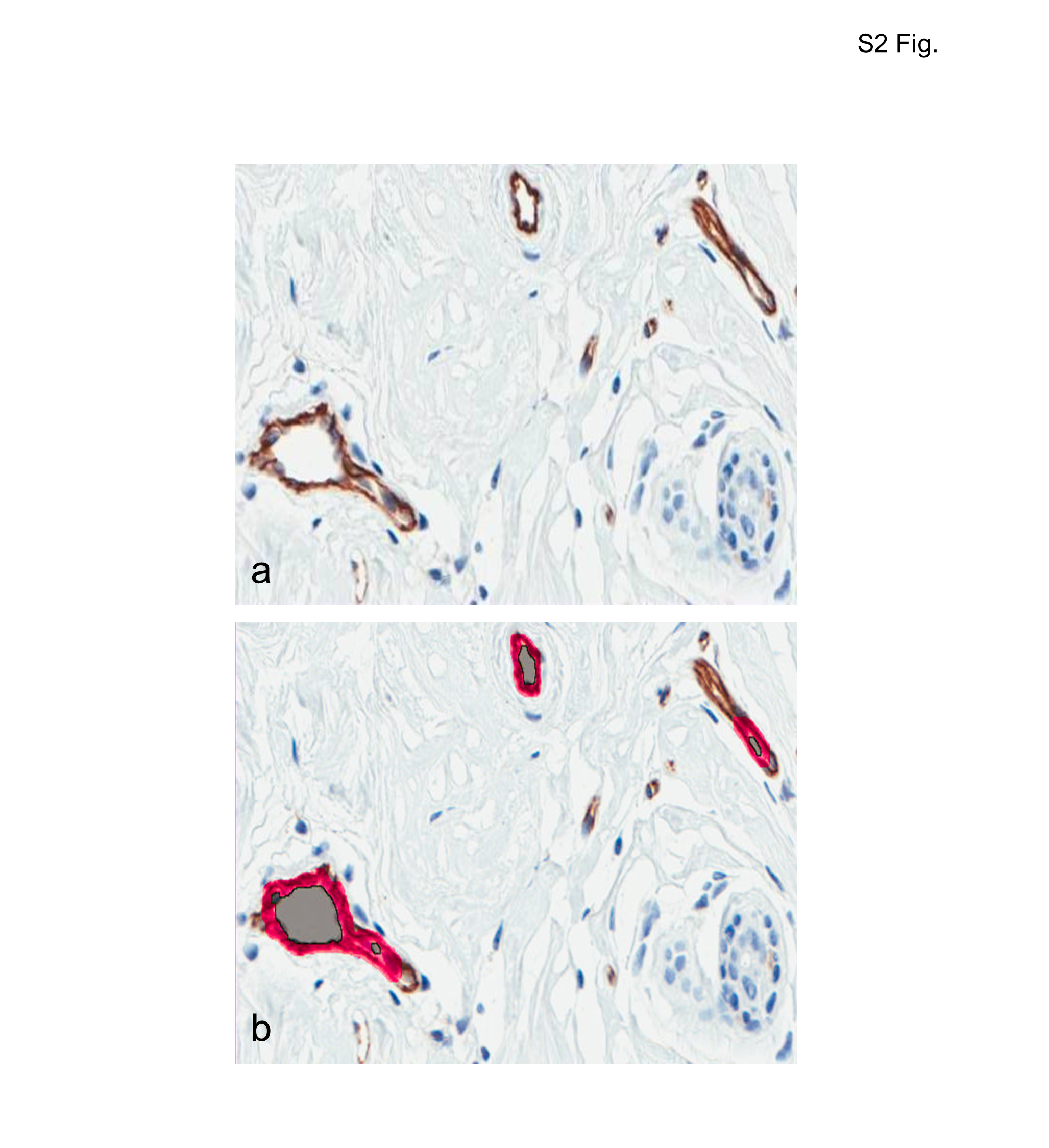

Supplement: S2 Fig — a) CD31 IHC-stained section; b) marked-up image of panel a with vascular luminal area (gray) and endothelial cells (red) highlighted (TIF) [file pone.0128861.s005.tif]

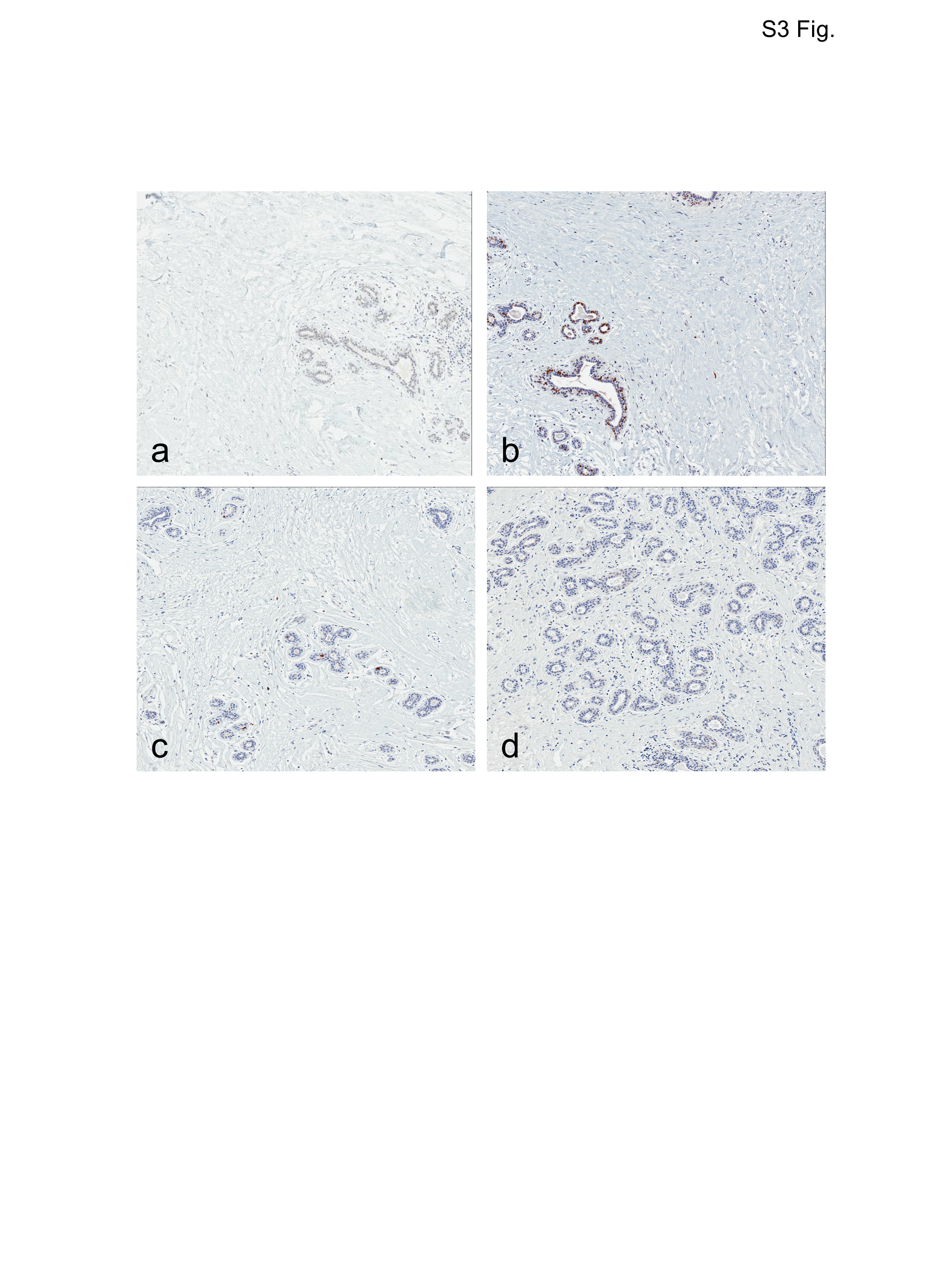

Supplement: S3 Fig — a) ERβ, b) PgR, c) Ki-67, d) HER2. (TIF) [file pone.0128861.s006.tif]
