# Supplementary material for: Breast Tissue Composition and Immunophenotype and Its Relationship with Mammographic Density in Women at High Risk of Breast Cancer
Source: PLoS One. 2015 Jun 25;10(6):e0128861. doi: 10.1371/journal.pone.0128861 (PMC4481506; doi:10.1371/journal.pone.0128861)
Supplement: S1 Supplementary Methods — (DOCX) [file pone.0128861.s007.docx]

**S1 Supplementary methods**

**Immunohistochemistry staining methods**

Sections (3μm thick) were cut from FFPE blocks, dewaxed, and hydrated through graded ethanol and water. Immunohistochemical stains for ERα, ERβ, PgR, and HER2 were performed as previously described ^12^.

For ERβ, antigen retrieval was performed in a pressure cooker in the high pH EnVision ^TM^ FLEX Target Retrieval Solution (Dako, Glostrup, Denmark) at 124^o^C with pressure set at 15 - 16 PSI for 4 minutes. Endogenous peroxidase activity in the cells was quenched with EnVision ^TM^ FLEX Peroxidase-Blocking Reagent (Dako) for 5 minutes at room temperature. The primary antibody, ERß1 antibody (mouse PPG5/10, GeneTex), was applied to the section at 1/15 and was incubated at 4^o^C overnight. The section was then incubated in EnVision^TM^ FLEX/HRP (Dako) detection system for 30minutes at room temperature. Freshly prepared 3’-diaminuteobenzidine (DAB; EnVision^TM^ FLEX DAB + Chromogen in EnVision^TM^ Flex substrate buffer, Dako) was applied to the sections and incubated at room temperature until a suitable intensity of DAB staining had developed.

Staining for ERα, PgR, HER2, CD31 and Ki-67 were performed on a Ventana BenchMark Ultra (Roche Diagnostics, USA). Antigen retrieval was performed in a high pH Ultra cell conditioning solution (CC1, Roche Diagnostics) for 36, 36, 20, 36 minutes and 52 minutes, respectively, followed by incubation with the antibody (ERα (SP1, Ventana, pre-diluted), PgR (1E2, Ventana, pre-diluted), HER2 (4B5, Ventana, pre-diluted), CD31 (JC7OA, Dako, diluted at 1/100), Ki-67 (SP6, Cell Marque, diluted at 1/50), at 36oC for 8, 12, 16, 36 and 32 minutes, respectively. Amplification kit (amplifiers A and B, Roche Diagnostics) and UltraView Universal DAB detection kit (Roche Diagnostics) were used in accordance with the manufacturer’s instructions for on-board detection.

All sections were counterstained with haematoxylin, blued in Scott’s, dehydrated in ethanol, cleared in xylene and mounted in Pertex.

**Image analysis**

Haematoxylin and eosin (H&E) stained slides (3µm-thick sections) were scanned using ScanScope XT (Aperio, Vista, CA, USA) at 20x magnification. The scanned slides were analyzed for tissue composition using the Positive Pixel Count (version 9) image analysis tool (Aperio, Vista, CA, USA), which counts the number of pixels showing positive and negative staining (Fig. 1). The positive stain colour was defined as a hue value of 0.93 (red-purple), with the range of positive colour detection set at a hue width of 0.5. The thresholds for strong, medium, and weak positive staining were at default settings. These parameters were chosen after optimization of the algorithm on H&E-stained sections of archival non-lesional breast tissue from non-study patients. The tissue section was then manually outlined by a specialist pathologist (JP). From the markup images generated, the strongly positive areas corresponded to epithelium, the moderately positive and weakly positive areas to fibrous stroma and the negative areas to fat. The number of strongly staining pixels (epithelium), moderate or weak staining pixels (stroma), or negative staining pixels (fat) was then used to calculate the proportion of each tissue type from the total number of pixels in the section (Fig. 1). In three cases, the fibrous stroma and epithelium was unable to be distinguished in this manner as there was altered staining due to diathermy artifact (one case), folded tissue section (one case), or prominent extravasated red blood cells (one case). In these cases, the epithelial and stromal areas were each manually outlined and quantified separately.

Vascular area was assessed from whole sections immunohistochemically stained for CD31 (Clone JC70A, Dako). These slides were scanned at 20x magnification using ScanScopeXT (Aperio, Vista, CA, USA) and analysed using the Microvascular Analysis Tool, version 1 (Aperio, Vista, CA, USA) which detects and quantifies microvessels on sections immunohistochemically stained for endothelial markers. The analysis area was defined by manually outlining the tissue in the section and the ‘Lumen and Vascular Cells’ analysis option was selected at default settings to generate markup images. The total stained area of the markup images (comprising the vascular lumen in addition to the surrounding endothelial cells) was then used to calculate the percentage of vascular area from the total analysis area. (S2 Fig.)

The immunohistochemically-stained slides were scanned at 20x magnification (ScanScopeXT, Aperio, CA, USA). ERα, PgR, and Ki-67 nuclear staining in the epithelium and stroma was analysed using the Nuclear (version 9) image analysis tool (Aperio, CA, USA) with parameters at default settings. ERβ nuclear staining in the epithelium and stroma was analysed using the Nuclear (version 9) image analysis tool (Aperio, CA, USA) with minimum nuclear size set at 10µm^2^, maximum nuclear size set at 60µm^2^ and intensity segmentation type selected. All other parameters were at default settings. These parameters were chosen after optimization of the algorithm on immunohistochemically-stained sections from non-study cases. Epithelial staining was assessed by manually outlining epithelial areas, and the number of positive staining nuclei (of any intensity) and the total number of nuclei were used to calculate the percentage of nuclei staining in the outlined area (Fig. 2). Stromal staining was assessed by manually outlining stromal areas, excluding epithelium, areas of confluent fat, and manually excluding areas of non-specific staining and cytoplasmic staining of stromal mast cells. The density of positive-staining nuclei was expressed as number of positive-staining nuclei (of any intensity) per mm^2^ of stroma (Fig. 2). Epithelial HER2 staining was scored as being either present or absent.
